# 3D-Web-GIS RFID Location Sensing System for Construction Objects

**DOI:** 10.1155/2013/217972

**Published:** 2013-06-19

**Authors:** Chien-Ho Ko

**Affiliations:** Department of Civil Engineering, National Pingtung University of Science and Technology, 1 Shuefu Road, Neipu, Pingtung 912, Taiwan

## Abstract

Construction site managers could benefit from being able to visualize on-site construction objects. Radio frequency identification (RFID) technology has been shown to improve the efficiency of construction object management. The objective of this study is to develop a 3D-Web-GIS RFID location sensing system for construction objects. An RFID 3D location sensing algorithm combining Simulated Annealing (SA) and a gradient descent method is proposed to determine target object location. In the algorithm, SA is used to stabilize the search process and the gradient descent method is used to reduce errors. The locations of the analyzed objects are visualized using the 3D-Web-GIS system. A real construction site is used to validate the applicability of the proposed method, with results indicating that the proposed approach can provide faster, more accurate, and more stable 3D positioning results than other location sensing algorithms. The proposed system allows construction managers to better understand worksite status, thus enhancing managerial efficiency.

## 1. Introduction

The construction industry is characterized by intensive manual labor and is prone to errors [1], creating significant challenges for providing a clear understanding of construction site activity [2]. A perennial issue facing construction site managers is object positioning including assets, personnel, material, and equipment [3–6]. Several attempts have been made to facilitate the location of objects on construction sites. Meade and Chignell [7] used ground penetrating radar to locate buried piping without excavation. Grau [8] presented an epistemic model based on belief functions to monitor the positions of mobile sensing nodes. His research demonstrates that the epistemic functions can correctly filter location uncertainties and effectively monitor the movements of mobile sensing nodes. Song et al. [9] tracked construction material to improve project performance and reduce the effort needed to derive project performance indicators. In their approach, materials are fitted with radio frequency identification (RFID) tags to allow for automatic identification and tracking on construction sites. Razavi and Moselhi [10] developed a construction equipment and supply location system using passive RFID tags. Skibniewski and Jang [11] introduced an architecture for construction asset tracking using wireless sensor modules to track objects via the time-of-flight method. Shahi et al. [12] presented an Ultra Wide Band positioning system as a material and activity tracking tool for indoor construction projects. Global Positioning System (GPS) is frequently used for tracking objects outdoors. Pradhananga and Teizer [13] used GPS devices to automate the assessment of construction site equipment operations by continuously logging time-stamped equipment locations for analysis. 

Given the alternatives available for object positioning in construction sites, Jiang et al. [14] and Nasir et al. [15] suggested methodologies for selecting appropriate technologies for various types of projects and objects and suggested that RFID is an appropriate solution for object positioning in indoor construction sites. Razavi and Moselhi [10] also demonstrated the potential for RFID as a method for object tracking in indoor construction sites. 

Accurate object positioning offers the possibility of improved object visibility. However, previous studies have either treated positioning algorithms as separate from display systems or have tended to use one- or two-dimensional maps to indicate object locations, thus obscuring object visibility. Displaying objects in three-dimensional (3D) space requires a corresponding location sensing algorithm. Ko [16] proposed an RFID 3D location sensing algorithm, but the algorithm had trouble deriving smooth convergences while searching for the target objects. 

The present study develops a 3D-Web-GIS RFID location sensing system to locate objects in indoor construction sites. An improved RFID 3D location sensing algorithm is established, combining Simulated Annealing (SA) and the gradient descent method to overcome the convergence problem while locating objects. The 3D-Web-GIS RFID location sensing system manipulates the location sensing algorithm to better relate the display of construction objects to the real world and help managers better understand construction site activity. This study begins by introducing indoor sensing networks and then explains the evolutionary process of the proposed RFID 3D location sensing algorithm. Section four describes the development of the 3D-Web-GIS RFID location sensing system. Section five describes a demonstration of the system on a construction site. Finally, the paper concludes with suggestions for future research directions.

## 2. Indoor Sensing Networks

Construction sites require both indoor and outdoor sensing [17, 18], but these sensing contexts require different types of networks and technologies [19]. GPS, a relatively mature location sensing technique, is frequently used in outdoor construction sites [20], but indoor location remains a challenge. This study thus focuses on improving the accuracy and efficiency of the location of indoor construction objects including material, equipment, personnel, and machinery. Construction site activity can only be understood through the simultaneous tracking of multiple objects. In a passive location system, RFID antennas are distributed at reference coordinates within the location space [21], and the target objects are fitted with passive RFID tags [22]. This provides a relatively low-cost solution as compared to active RFID systems in which the target objects have to be equipped with antennas. A passive location mode that attaches an active RFID tag on the target object [23] is therefore selected. 

**Figure 1:**
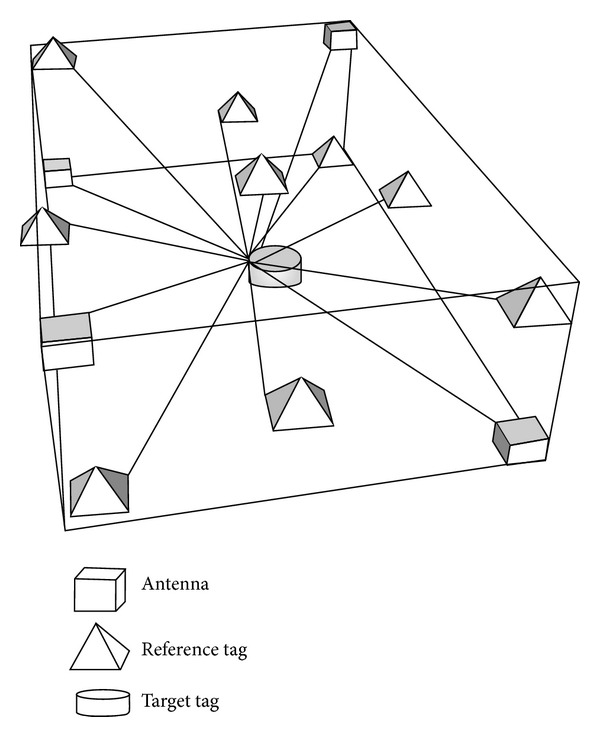
Indoor sensing networks.

The indoor sensing networks are constructed using RFID readers and tags, as shown in Figure 1. An active RFID system was used to expand the sensing space. Four RFID antennas were set at four corners of the hexahedron space. Nine reference tags were uniformly distributed in the space to build a location sensing network. Through the network topology, signal strengths of the RFID tags from different directions with diverse distances can be collected. The collected signal strength was then analyzed using a 3D location sensing algorithm to calculate target object locations. 

## 3. Location Sensing Algorithms

### 3.1. Location Concept

**Figure 2:**
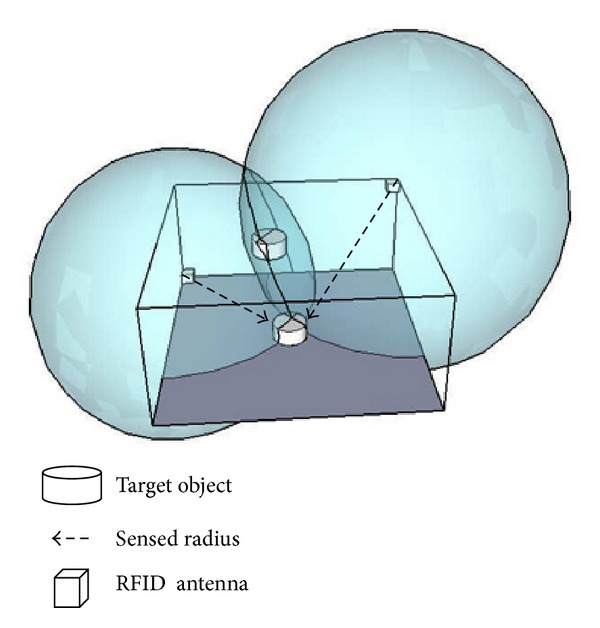
3D trilateration location concept.

This research develops an RFID 3D location sensing algorithm using a trilateration method [24–26] that calculates the target object location using distances from the RFID antennas to the target object. In a 3D space, a single RFID antenna can sense its distance to an RFID tag using Received Signal Strength Indication (RSSI). The possible location of the target object could be expressed as a sphere with a radius of the sensed distance. Adding a second antenna, the solution space is an intersection of the two spheres with the two sensed radii, as shown in Figure 2. Using the same concept, the target object location could be further narrowed to few points using three antennas. Four antennas can be expected to produce a highly specific location. 

### 3.2. Location Algorithm

**Figure 3:**
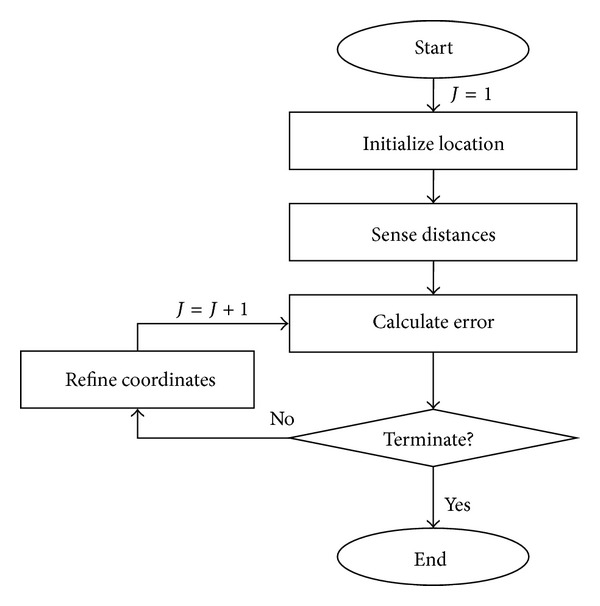
RFID 3D location sensing algorithm.

Ko [16] developed an RFID 3D location sensing algorithm using the gradient descent method. However, that method used fixed adjustment coefficients to search for the target object location. In a large space, the adjustment coefficient has to be increased to reduce the amount of computational time required, but this may make convergence difficult. A small space, on the other hand, needs smaller adjustment coefficients to converge. Selection of the adjustment coefficients appropriate for the dimensions of the given search space is achieved by trial and error. Simulated Annealing (SA), a technique analogizing the annealing of metals for stable global search [27], could potentially solve this problem. This study thus hybridizes SA and gradient descent methods to locate construction-related objects in a 3D space. The improved RFID 3D location sensing algorithm is shown and explained in Figure 3. 

#### 3.2.1. Initializing Location

The first step of the algorithm is to initiate a location search for the target object. The location of target object *i* in 3D space is noted as (*x*_*i*_, *y*_*i*_, *z*_*i*_). 

#### 3.2.2. Sensing Distances

**Figure 4:**
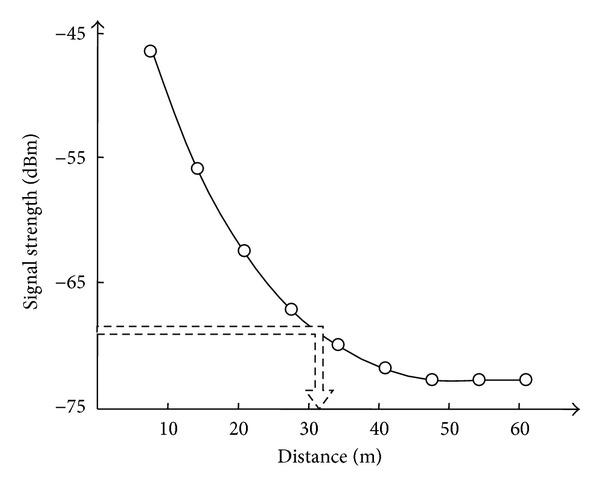
Received signal strength indication.

The trilateration location method requires the distances from each antenna to the target object. The proposed method senses the distance using RSSI, with an example shown in Figure 4. An antenna receives a signal with a given strength level from the active RFID tag attached to the target object. Through the RSSI curve, the received signal strength can be converted to a distance. 

#### 3.2.3. Calculating Error

This step calculates a positioning error between the initial location and the sensed location, which will be used to adjust the target object location in the next step. The positioning error of target object *i* and antenna *k* (*e*_*ik*_) is calculated using the following equation:

(1) $$\begin{array}{c} e_{ik}=\left(S_{ik}-{\bar{S}}_{ik}\right), \end{array}$$

where *S*_*ik*_ is a sensed distance between target object *i* and antenna *k* converted using RSSI; ${\bar{S}}_{ik}$ is the distance in 3D space between target object *i* at (*x*_*i*_, *y*_*i*_, *z*_*i*_) and antenna *k* at (*x*_*k*_, *y*_*k*_, *z*_*k*_) calculated using the following equation:

(2) $$\begin{array}{c} {\bar{S}}_{ik}=\sqrt{{\left(x_{i}-x_{k}\right)}^{2}+{\left(y_{i}-y_{k}\right)}^{2}+{\left(z_{i}-z_{k}\right)}^{2}}. \end{array}$$

#### 3.2.4. Refining Coordinates

SA is combined with the gradient descent method to narrow the potential location of the target object. SA is used to gradually decrease the adjustment (i.e., cooling down) to help the algorithm converge, while the gradient descent method is used to reduce location error. The target object's location at the epoch (*j*) is adjusted using the following equation:

(3) (xi(j+1),yi(j+1),zi(j+1))  ={xi(j+1)=xi(j)+Δxi(j)yi(j+1)=yi(j)+Δyi(j)zi(j+1)=zi(j)+Δzi(j),

where (Δ*x*_*i*_(*j*), Δ*y*_*i*_(*j*), Δ*z*_*i*_(*j*)) is the amount of adjustment for the *x*-, *y*-, and *z*-axes. SA is applied to cool down the adjustment, as shown in the following equation:

(4) $$\begin{array}{c} \left(\Delta x_{i}\left(j\right),\Delta y_{i}\left(j\right),\Delta z_{i}\left(j\right)\right)=\{\begin{array}{c} \Delta x_{i}\left(j\right)=\alpha_{x}\beta_{x}\gamma_{x}\\ \Delta y_{i}\left(j\right)=\alpha_{y}\beta_{y}\gamma_{y}\\ \Delta z_{i}\left(j\right)=\alpha_{z}\beta_{z}\gamma_{z}, \end{array} \end{array}$$

where (*α*_*x*_, *α*_*y*_, *α*_*z*_) is adjustment rate for the *x*-, *y*-, and *z*-axes; (*β*_*x*_, *β*_*y*_, *β*_*z*_) represents the temperature formulated in (5); (*γ*_*x*_, *γ*_*y*_, *γ*_*z*_) is the cooling speed represented in (6). Consider

(5) $$\begin{array}{c} \left(\beta_{x},\beta_{y},\beta_{z}\right)=\{\begin{array}{c} \beta_{x}=\frac{\left(S_{ik}-{\bar{S}}_{ik}\right)}{\left|S_{ik}-{\bar{S}}_{ik}\right|}\exp\left(\frac{-1}{\alpha_{x}\left|x_{i}\delta_{ik}\right|}\right),\\ \beta_{y}=\frac{\left(S_{ik}-{\bar{S}}_{ik}\right)}{\left|S_{ik}-{\bar{S}}_{ik}\right|}\exp\left(\frac{-1}{\alpha_{y}\left|y_{i}\delta_{ik}\right|}\right),\\ \beta_{z}=\frac{\left(S_{ik}-{\bar{S}}_{ik}\right)}{\left|S_{ik}-{\bar{S}}_{ik}\right|}\exp\left(\frac{-1}{\alpha_{z}\left|z_{i}\delta_{ik}\right|}\right), \end{array} \end{array}$$

(6) $$\begin{array}{c} \left(\gamma_{x},\gamma_{y},\gamma_{z}\right)=\exp\left(\mu \right). \end{array}$$

In (5), the *k* RFID antenna gradient for target *i* (*δ*_*ik*_) can be calculated using (7). The *μ* shown in (6) is a parameter simulating the cooling process, which is formulated using (8). Consider

(7) $$\begin{array}{c} \delta_{ik}={\bar{s}}_{ik}\times e_{ik}, \end{array}$$

(8) $$\begin{array}{c} \mu =-\;0.2\times \left(j-1\right). \end{array}$$

The *k* RFID antenna adjusts the target object's location using (3) to (8). The error of the target object *i* for *m* antennas (*ε*_*i*_) is calculated using the Root Mean Square Error (RMSE), as shown in the following equation:

(9) $$\begin{array}{c} \varepsilon_{i}=\sqrt{\frac{\sum_{k=1}^{m}{\left(\left(S_{ik}-{\bar{S}}_{ik}\right)/S_{ik}\right)}^{2}}{m}}. \end{array}$$

#### 3.2.5. Terminating Conditions

The algorithm locates the target object's location using an iterative adjustment process. The termination conditions can be met if epoch number (*j*) reaches a predetermined criterion and/or the RMSE (*ε*_*i*_) is smaller than an assigned number. The predetermined epoch number can ensure that the location algorithm is completed within a specified duration, while the preassigned RMSE ensures the location's accuracy. 

## 4. System Development

### 4.1. Use Case

**Figure 5:**
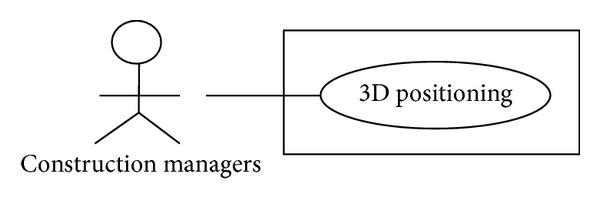
Use Case diagram.

The Rational Unified Process (RUP) [28] and Unified Modeling Language (UML) [29] were used to develop the 3D-Web-GIS RFID location sensing system. To identify system requirements, Use Case, which is regarded as a high-level system descriptor, is used for system analysis and design.  Figure 5 shows the Use Case diagram of the 3D-Web-GIS RFID location sensing system. The diagram shows how the system can be used by construction managers to locate objects in construction sites and by project stakeholders to easily understand the system. 

The Use Case used in this study is explained as follows:

- Use Case: 3D positioning,
- actor: construction managers,
- type: primary,
- Descriptions:
  1. users select an object, and the system then displays the object's location in 3D space,
  2. users click the displayed object to retrieve information about the object.

**Figure 6:**
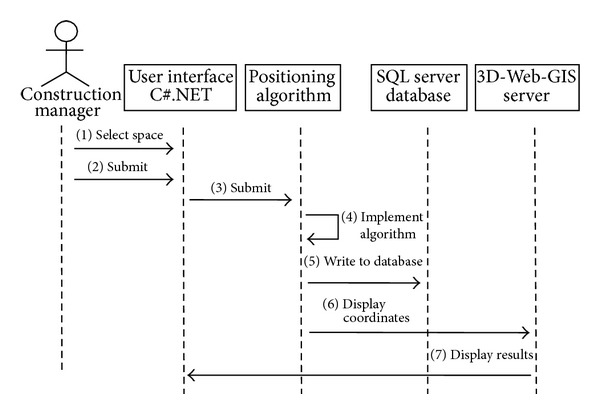
Positioning sequence diagram.

**Figure 7:**
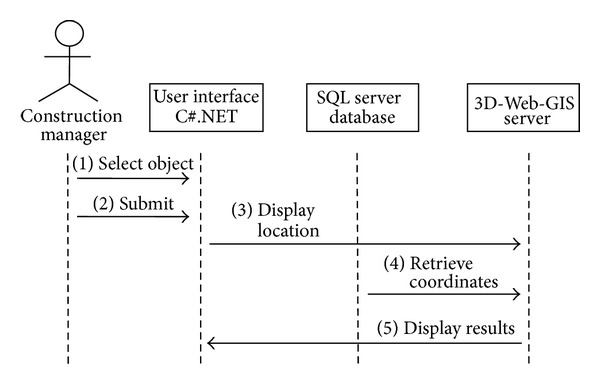
Browsing sequence diagram.

The Use Case identifies two system functions: position and browse. These functions are explained in Table 1, while their sequence diagrams are shown in Figures 6 and 7.

### 4.2. System Architecture

**Figure 8:**
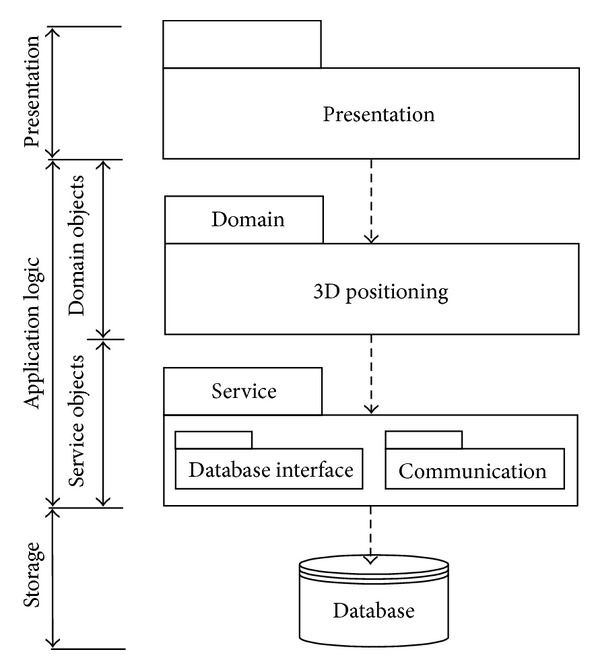
System architecture.

Figure 8 displays the architecture of the 3D-Web-GIS RFID location sensing system in three tiers. Location sensing algorithm parameters and object coordinates are stored in the storage layer. The 3D positioning algorithm is in the application logic layer that implements system functions. The presentation layer provides user interfaces allowing users to interact with the application logic layer. The system was developed using Microsoft Visual Studio. Net (C#.NET) with SQL server database. Construction object geographic information is displayed by integrating an ESRI ArcGIS Server with the 3D extension module via C#.NET. 

## 5. Verification

To validate its feasibility, the proposed algorithm was applied to a real construction site: a 926 cm × 535 cm × 211 cm indoor space on the third floor of a construction site. The target object is located at (694,400,75). An adjustment rate of (0.5) is used for (*α*_*x*_, *α*_*y*_, *α*_*z*_). 

**Figure 9:**
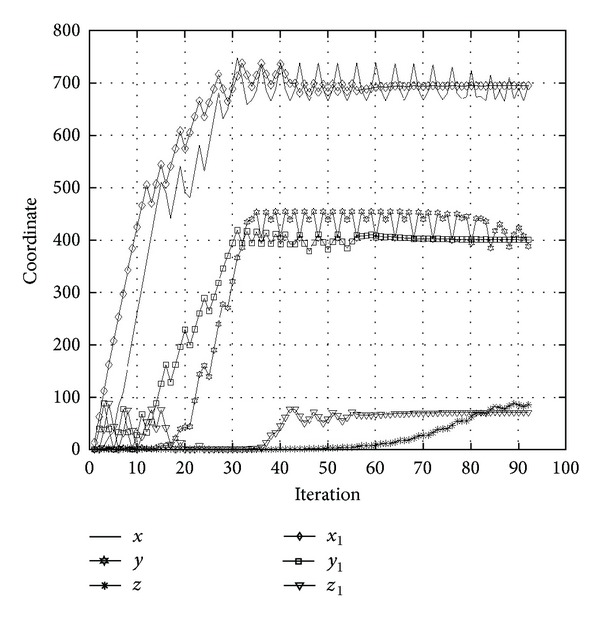
Location convergence comparison.

**Figure 10:**
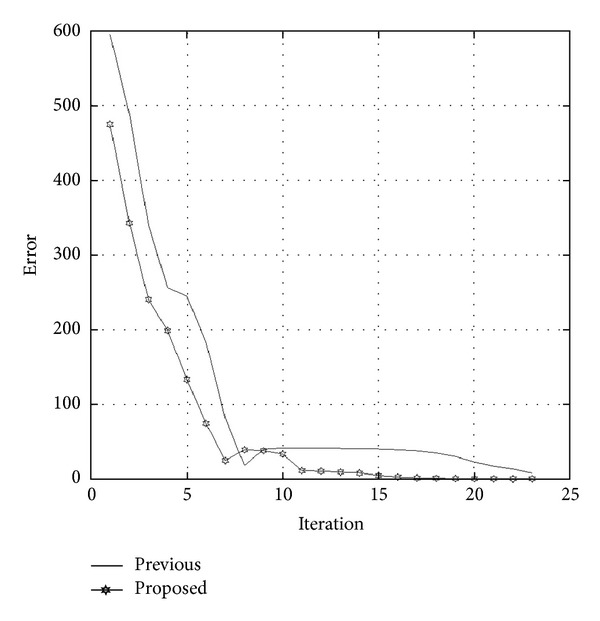
Error convergence comparison.

**Figure 11:**
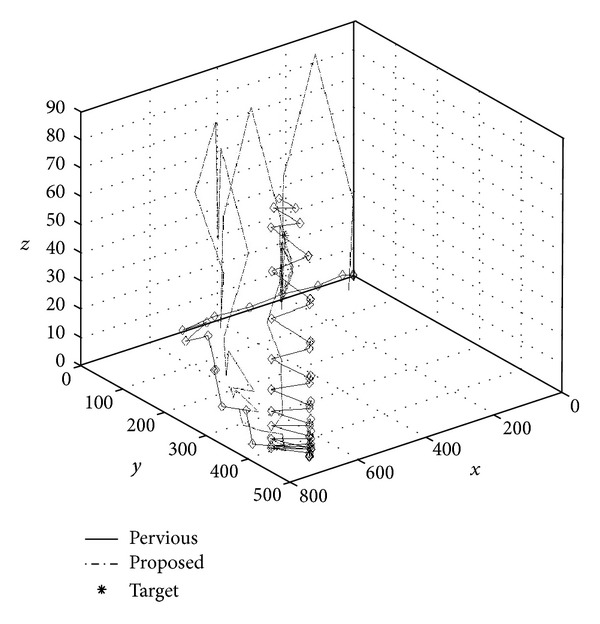
3D locus comparison.

**Figure 12:**
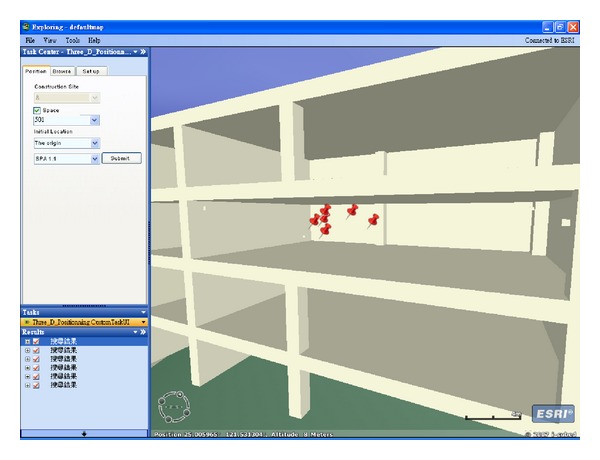
Target object location display.

**Figure 13:**
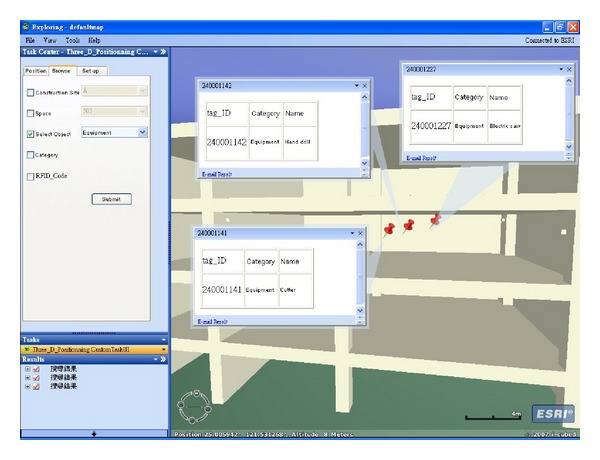
Browsing construction object information.

Setting the initial location of the target object at (1,1, 1), Figure 9 compares the convergence trend of the proposed method with that developed by Ko [16]. In the figure, *x*, *y*, and *z* are convergence trends of the previous method, while *x*_1_, *y*_1_, and *z*_1_ are those of the proposed method. The proposed method locates the target object at iteration 60, as opposed to iteration 90 for the previous method. The previous method adjusts the target object's location using gradient decent method. Thus, although adjustments move in the right direction, the convergence becomes spiky in later stages. By contrast, the proposed method combines SA and the gradient decent method to adjust the target object's location. In the early stages of positioning, both methods display the same conspicuous adjustments. In the cooling down stage, smoother adjustments are applied to locate the target object.  Figure 10 compares the error convergence between the two methods.  Figure 11 shows the locus of the two methods in 3D space while positioning the target object. As discussed, the proposed method locates the target object faster, more accurately, and more stably in 3D space. Finally, the location of the target object is displayed using the developed 3D-Web-GIS system, as shown in Figure 12, and the location of the construction objects can be visualized. By clicking the located objects, object information is retrieved from the database, as shown in Figure 13. 

## 6. Conclusions

This study hybridizes SA and the gradient descent method to develop a 3D RFID location sensing algorithm. A 3D-Web-GIS system is developed to run the algorithm and display the location of target objects. The proposed method is validated by application to a real construction site, with performance comparisons to the previous best method. 

In the proposed algorithm, SA is used to stabilize the search process while the gradient descent method is used to increase location accuracy. At the beginning of the search, while the temperature is high, large location adjustments are made, thus saving time for positioning. The temperature gradually cools as the positioning process enhances the convergence. Application to a real construction site validates that combining SA with the gradient descent method improves the speed, accuracy, and stability of results over those obtained using the previous location sensing algorithm in 3D positioning. Furthermore, the previous 3D positioning algorithm needs to determine algorithm parameters according to location space dimension. The proposed positioning algorithm, however, frontloads target object searching to the beginning of the search process and gradually cools down over time and thus may not need to predetermine algorithm parameters due to the size of the spatial dimensions. 

The developed 3D-Web-GIS RFID location sensing system can be accessed through the Internet. Positioning results are visualized in 3D environment, and users can browse information related to the located construction objects. The system allows construction managers to locate construction objects at any time, from anywhere using any operating systems, thus enhancing managerial efficiency. 

This study did not take into account the RFID signal attenuation effect caused by environmental factors, and future studies could modify the proposed algorithm to consider signal attenuation. Future work could also use this 3D location sensing technology to develop mechanisms for tracking the movement of construction objects within construction sites. 

## Figures and Tables

**Table 1:** Location sensing system functions.

Function	Explanation	Category
Position	Users select a construction site and space. The system calculates and displays object locations using the 3D-Web-GIS system.	Evident

Browse	Users select a construction-related object. The system displays information about the selected object using the 3D-Web-GIS system.	Evident
